# Direct and Inverse Correlates of Post-Traumatic Stress Disorder among School-Age Autistic Boys

**DOI:** 10.3390/ijerph18105285

**Published:** 2021-05-16

**Authors:** Vicki Bitsika, Christopher F. Sharpley

**Affiliations:** Brain-Behaviour Research Group, School of Science and Technology, University of New England, Armidale, NSW 2350, Australia; vicki.bitsika@une.edu.au

**Keywords:** autism, trauma, stress, bullying, school

## Abstract

Young people with autism are often bullied at school, a potential direct correlate of Post-Traumatic Stress Disorder (PTSD). This may be compounded by their difficulties in social interaction. Alternately, some of these young people may develop ‘coping strategies’ against bullying that may have an inverse association with PTSD. As a vulnerable population for PTSD, a sample of 71 young males with autism were surveyed for their self-reported experiences of being bullied at school, their coping strategies for dealing with this bullying, and their own evaluations of the severity of two of the key diagnostic criteria for PTSD. Their mothers also provided a rating of the severity of the three major diagnostic criteria for autism for these boys. Over 80% of this sample had been bullied, and there was a significant direct correlation between this and PTSD score, and between their mother-rated severity of the boys’ social interaction difficulties, but also a significant inverse correlation between their coping strategies and PTSD score. There were differences in these relationships according to whether the boys attended elementary or secondary school. These findings hold implications for the identification, assessment and support of autistic youth at risk of PTSD.

## 1. Introduction

Post-Traumatic Stress Disorder (PTSD) is most commonly studied in specific populations that have undergone some major stressful event. For example, in a meta-analysis of 11 studies of 1532 children and adolescents who had experienced a road traffic accident, Dai et al. [1] found that the pooled prevalence of PTSD was 19.95%. Even higher prevalence was reported for young people who had experienced an earthquake six months prior to assessment (58.3%: [2]). One population that is vulnerable to stress, anxiety, depression and PTSD is young autistic people (ASD) [3,4,5,6] (We follow the preferences of autistic people in this nomenclature [7]).

One of the defining characteristics of ASD is difficulties in social interaction and communication [8], which can be a source of great stress to autistic youth [9]. As the progression from stress to anxiety to PTSD has been established [10], it is of potential value to identify some of the possible ‘triggers’ for PTSD in autistic youth, particularly those environmental events that are associated with social interaction and communication. One such event is being bullied at school, which has been linked to anxiety and depression in autistic youth [11].

As well as the longer-term effects of an almost doubled likelihood of requiring psychiatric treatment during adulthood due to having been bullied while a child (Sourander et al., 2016), qualitative studies show that being bullied in school is extremely stressful for autistic youth (Goodall, 2018), and can be associated with a refusal to attend school [12]. When the finding that nearly half (44%) of school-age autistic youth report being bullied [13], particularly at school [14], the potential importance of being bullied at school as a potential contributor to PTSD is clear for autistic youth.

Haruvi-Lamdan, Horesh, and Golan [15] argued that little had been reported regarding the association between being bullied at school and PTSD in autistic youth, arguing “a pressing need for more PTSD-ASD research”, focusing on the effects of social events, such as being bullied. This study aimed to investigate that issue via four research questions: (1) what is the relative prevalence of being bullied and PTSD in a sample of autistic youth; (2) is there a significant correlation between these two factors, and between the major diagnostic criteria for ASD; (3) do autistic youth exert coping strategies against being bullied; and (4) how effective are those coping strategies? The answers to these questions have the potential to inform clinical practice by identifying the extent of the association between bullying and PTSD, as well as assisting bullied autistic youth to develop effective coping strategies.

Although a formal diagnosis of PTSD requires the presence of a number of symptoms including exposure to a traumatic event, distressing memories and/or dreams of the traumatic event, avoidance of stimuli associated with the traumatic event, negative changes in cognition or mood, and increased arousal, there is a great deal of variety in how PTSD presents [8]. In children and adolescents, the two key diagnostic criteria are (a) experience of an extremely upsetting event (either directly or observed), plus (b) re-experiencing of that event, most commonly via dreams or memories [8]. By contrast, avoidance of the traumatic event, changes in cognition or mood, and increased arousal, may only occur when the individual is exposed to the stimuli associated with the event itself [8]. Screening of PTSD may therefore be accomplished in youth participants via the application of the two diagnostic criteria listed under (a) and (b) above. 

Because of recent reports that social communication difficulties may produce different responses in autistic males versus females [16], the potential confound arising from sex of participant was constrained by selecting only males as participants in this study2.

## 2. Materials and Methods

### 2.1. Participants

Seventy-one autistic boys and their mothers responded to a call for participation made to several parent organisations on the Gold Coast, Australia (no attempt was made to restrict the parents to just mothers). The boys were aged 7 years to 18 years (*M* = 11.63 years, SD = 2.42 years). They all attended mainstream schools, in grades two to six (elementary school), and seven to 11 (secondary school), and had an Intelligence Quotient (IQ) of at least 70 on a Wechsler individualised test of IQ, administered during their initial assessment for ASD. They all also had received a formal diagnosis of ASD from a psychiatrist or paediatrician, which was confirmed by a suitably-qualified psychologist. Administration of the Autism Diagnostic Observation Schedule (ADOS) or Autism Diagnostic Interview-Revised (ADIR) is not required in Australia for the purposes of a diagnosis of ASD.

### 2.2. Measures

Questionnaire. Mothers completed a questionnaire about their son’s age, and his difficulty in regards to: socialising, communicating, and restricted and repetitive behaviours (the key diagnostic criteria for ASD) by rating the boys’ difficulties as ‘minimal’, ‘slight’, ‘moderate’, ‘severe’, or ‘very severe’ (corresponding to a score of 1, 2, 3, 4 or 5)..The sons completed a questionnaire about whether they had been bullied (yes/no), frequency of being bullied (not often, sometimes, nearly every day), and what strategies they had used to deal with the bullying they received (*ignore the bullies*, *smile at them*, *say something back to them*, *hit or push or kick*, *chase them away*, *walk away*, *avoid being near them*). The sons were also asked to respond to the CASI-4 PTSD subscale (described below).

Child and Adolescent Symptom Inventory, 4th Revision (CASI-4R). The CASI-4R [17] is a screening test based upon Diagnostic and Statistical Manual of Mental Disorders, 4th revision, Text revision (DSM-IV-TR) criteria for a range of disorders. It was normed in a variety of samples, including studies of 103 [18] and 67 autistic children [19]. Psychometric data are described in the CASI-4 Test Manual [17,20], and include a test–retest reliability of *r* = 0.67 (*p* < 0.001) over a six-week period, internal consistency of 0.74, strong criterion-related validity with psychiatric diagnoses, internal construct validity, and discriminant validity [17]. Responses to the CASI-4R questionnaire items range from 0 (never), 1 (sometimes), 2 (often), or 3 (very often) for items representing diagnostic criteria.

CASI-4R PTSD subscale. The CASI-4R has several subscales, including one that screens for PTSD, consisting of two items derived from the DSM-IV-TR diagnostic criteria for PTSD: *has experienced an extremely upsetting event and continues to be bothered by it*, and *has distressing memories or dreams about an extremely upsetting event*. The combined score from these two items was named the ‘CASI-4R PTSD score’, or ‘PTSD score’ in this study, to signify that it did not represent a formal diagnosis of PTSD but rather an indication of the likely presence of PTSD.

Source of ratings. Autistic children can self-evaluate their social interaction strategies [21] and their own emotions [22] including loneliness [23], anxiety [24], and depression [25]. Previous research has indicated that data regarding autistic boys’ anxiety can be collected from the boys themselves using the CASI-4R [26], and there are some data arguing that there is a stronger correlation between such self-ratings of anxiety and a biological indicator of chronic anxiety [27]. Hence, the self-rating of the sons’ PTSD score was used in this study.

### 2.3. Procedure

Ethical approval for this study was obtained from the Bond University Human Research Ethics Committee (BUHREC). All participants were informed that the responses they and their sons gave would be kept confidential. Participants were given an online address to access the questionnaire so that the mothers’ and sons’ data could be collected in tandem (though discretely from each other) and linked via the online data-collection system.

### 2.4. Statistical Analyses

Data were downloaded from the online data-collection service and analysed by SPSS version 25. The CASI-4R PTSD data were tested for normality. The principal statistical test used to detect associations between PTSD score and the boys’ ages, difficulties with key ASD diagnostic criteria, and coping strategies was Pearson or Spearman correlational analysis because sample size constraints restricted the use of regression analysis. *A priori* power analysis indicated that a sample of 50 participants would be sufficient to detect a medium strength correlation of 0.40 with *p* < 0.05 and power of 0.90. Analysis of Variance tested for differences in CASI-4R PTSD score between bullied and non-bullied boys, and between elementary and secondary school bullied boys. Where cell sizes were unequal, Levine’s test for equality of variances was checked and found to not indicate unequal variances.

## 3. Results

### 3.1. Sample Data

Internal consistency (Cronbach’s alpha) for the CASI-4R PTSD subscale was 0.769; inspection of the Normal Q–Q Plot revealed a straight line, which indicates a normal distribution, and so no transformation of the raw data was undertaken. 

Of the 71 boys, 58 (81.7%) reported that they had been bullied at school, and these boys had significantly higher CASI-4R PTSD scores (*M* = 2.121, SD = 1.855) than those boys who reported that they had not been bullied at school (*M* = 0.154, SD = 0.376; *F*(1,70) = 14.328, *p* < 0.001, *µ*^2^ = 0.172), which represents a large effect size [28]. There was no significant correlation between the bullied boys’ ages and their CASI-4R PTSD score (*r* = −0.138, *p* = 0.251), but the 41 bullied boys in elementary school had higher CASI-4R PTSD scores (*M* = 2.487, SD = 1.982) than the 17 bullied boys in secondary school (*M* = 1.235, SD = 0.976; *F*(1,57) = 5.954, *p* = 0.018, *µ*^2^ = 0.096), which was a moderate effect. The frequency of being bullied was not significantly correlated with the bullied boys’ CASI-4R PTSD score (*ρ* = 0.239, *p* = 0.090). Parents’ ratings of the severity of their son’s ASD diagnostic criteria for difficulties with socialising was significantly correlated with bullied boys’ CASI-4R PTSD scores (*ρ* = 0.379, *p* = 0.003) but the parents’ severity ratings of their bullied sons’ difficulties with communication (*ρ* = 0.182, *p* = 0.172) or difficulties with rigid or repetitive behaviours (*ρ* = 0.192, *p* = 0.148) were not significantly correlated with the bullied sons’ CASI-4R PTSD score.

The boys were asked to rate their use of a series of potential coping strategies for dealing with being bullied, described above in Methods. Spearman’s correlation analysis was used to determine if any of those strategies were significantly correlated with the bullied boys’ CASI-4R PTSD score. Table 1 presents the results of that analysis. Only one strategy was significantly and inversely correlated with the boys’ CASI-4R PTSD score (i.e., *saying something back to them*). The only other strategy that was close to being significantly correlated with CASI-4R PTSD score was *chasing them away*.

### 3.2. Elementary vs. Secondary School Boys

As mentioned above, although there was no significant correlation between age and CASI-4R PTSD score at the whole sample level, those bullied boys who were in elementary school had significantly higher PTSD scores than bullied boys in secondary school. However, this was the only significant difference in the variables examined here across the two school subgroups of bullied boys when tested via MANOVA (all *p* > 0.05). This result enabled investigation of the associations between the bullied boys’ CASI-4R PTSD scores, ASD-related variables (difficulties with socialising, communication, and rigid or repetitive behaviours) and their coping strategies, within each school subgroup.

**Figure 1:**
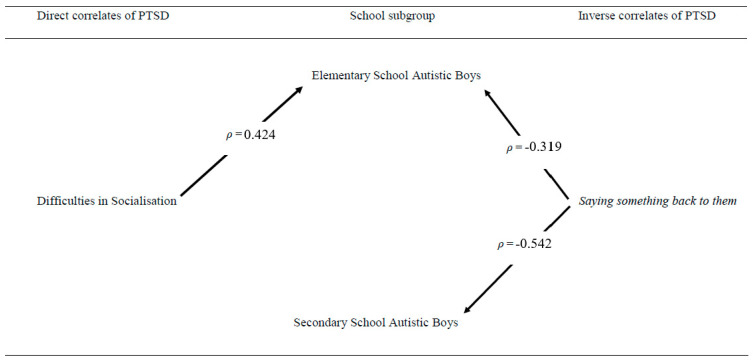
Significant direct and Inverse correlates of PTSD in Elementary and Secondary school autistic boys.

Correlational analysis revealed that the mothers’ ratings of their elementary school sons’ difficulties with socialisation were significantly directly correlated with their CASI-4R PTSD score (*ρ* = 0.424, *p* = 0.006), and the boys’ coping strategy of *Saying something back* to bullies was significantly inversely correlated with their CASI-4R PTSD score (*ρ* = −0.319, *p* = 0.042). However, only the secondary boys’ coping strategy of *saying something back to them* was significantly inversely correlated with their CASI-4R PTSD scores (*ρ* = −0.542, *p* = 0.025). Mothers’ ratings of their sons’ difficulties in socialisation were not significantly correlated with the boys’ PTSD score (*ρ* = 0.228, *p* = 0.379). None of the other ASD-related variables or the boys’ coping strategies were significantly correlated with the boys’ CASI-4R PTSD scores within either of these two school-defined subgroups. Figure 1 presents these results in graphic format.

## 4. Conclusions

Having been bullied was significantly associated with elevated PTSD scores on the screening subscale of the CASI-4R in this sample of autistic youth, supporting the hypothesis that these boys were vulnerable to this psychiatric disorder, although formal assessment would be required to confirm the presence of a full diagnosis of PTSD. This study did not set out to compare autistic and non-autistic boys for PTSD, which has been previously reported [6,29], but rather to investigate the direct and inverse correlates of the key diagnostic symptoms for PTSD.

There was no significant association between frequency of being bullied and the severity of the CASI-4R PTSD score, nor any significant association between the severity of two of the three diagnostic criteria for ASD, as perceived by the boys’ mothers. However, the mothers’ ratings of their sons’ difficulties in engaging socially with others was a significant direct correlate of the boys’ PTSD score severity. It has been suggested that being bullied is not a random event, and that the ASD trait of low social interaction skills may be a predictor of being bullied at school [30]. That hypothesis was confirmed in this study, for both elementary and secondary school autistic boys, and highlights the vulnerability of these boys to PTSD, simply because of their autism. 

Only one of the boys’ coping strategies was an inverse correlate of PTSD score—the act of verbally replying to bullies, and that was only significant for the elementary school boys. These findings suggest that, as well as having more severe CASI-4R PTSD scores, the elementary school boys also employed a coping strategy which may have provided them with some protection from the PTSD-inducing effects of being bullied, although verification of that effect requires longitudinal data There was a nonsignificant trend for another coping strategy to be associated with lower PTSD score in the whole sample—that of *Chasing them away*.

Although only one of these two strategies reached acceptable levels of statistical significance, these two strategies are similar in that they involve some response from the bullied autistic boys that could be described as ‘active’ by requiring the bullied autistic boy to confront the bully. The only other similarly confronting strategy was to *be physically aggressive*, which may have been unacceptable within the school environment. The remaining strategies were all “avoidant”, in that they entailed *ignoring the bully*, *smiling and acting as if the bullying was not offensive*, *walking away*, and *avoiding being near the bully*.

This study did not set out to evaluate the various coping strategies used by these boys in any conclusive manner, but rather to explore them as potential ways that these autistic boys might provide some self-protection against the traumatic effects of being bullied. This information may contribute to clinical settings by focussing training efforts for autistic boys to help them reduce the traumatic effects of being bullied. It has been reported that screening for trauma is undertaken and recommended in clinical practice with autistic youth [31], and the current results may help extend that process into potentially fruitful coping strategies for autistic boys.

This study has some limitations upon the generalisability of its results. Only autistic males were recruited to reduce the potential confound from sex effects, and replication with autistic girls would be valuable. All the autistic boys were ‘mildly impaired’ in that they had an IQ of 70 or more and were attending mainstream schools, so no conclusions can be drawn regarding autistic boys with lower IQ or greater autism impairment. The measure used (CASI-4R PTSD subscale) does not purport to provide a diagnosis of PTSD but is instead used as a screening device; formal psychiatric assessment of PTSD would enhance the validity of these findings. Data were collected via online participation, and no information is available regarding potential participants who did not accept the invitation to take part in this study. Finally, data were collected at a single time point, and so generalisability is limited to a cross-section of the lives of these autistic boys; similarly, without longitudinal data, no formal causal deductions can be drawn from these results.

Notwithstanding these caveats, this study extends the literature regarding the vulnerability of autistic boys to PTSD that is associated with being bullied at school. As noted in the introduction, nearly half of autistic youth report being bullied [13]. On the basis of the current findings that over 80% of the sample had been bullied, and that they also had elevated CASI-4R PTSD scores compared to their peers who had not been bullied, the possible association between being bullied and developing PTSD is of concern and argues for greater focus on the prevalence of both bullying and PTSD in autistic youth. Additionally, the finding that one of the key characteristics of autism was also a significant direct correlate of PTSD underlines the importance of assisting these boys in learning more appropriate social interaction skills. Their ability to confront their bullies is a potential target for building better strategies for avoiding PTSD.

## Figures and Tables

**Table 1:** Spearman correlations between seven coping strategies employed by 58 autistic boys who had been bullied and their CASI-4R PTSD score.

Strategy	Spearman Correlation with CASI-4R PTSD Score	*p*
Ignore them	0.007	0.961
Smile and act OK	0.054	0.686
Say something back to them	−0.370	0.004
Be physically aggressive	−0.145	0.278
Chase them away	−0.239	0.071
Walk away from them	−0.111	0.405
Avoid them	−0.041	0.759

## Data Availability

The data presented in this study are available on request from the corresponding author.

## References

[1] Dai W., Liu A., Kaminga A., Deng J., Lai Z., Wen S. (2018). Prevalence of Posttraumatic Stress Disorder among Children and Adolescents following Road Traffic Accidents: A Meta-Analysis. Can. J. Psychiatry.

[2] Marthoenis M., Ilyas A., Sofyan H., Schouler-Ocak M. (2019). Prevalence, comorbidity and predictors of post-traumatic stress disorder, depression, and anxiety in adolescents following an earthquake. Asian J. Pharm..

[3] Sharpley C., Bitsika V., Andronicos N., Agnew L. (2016). Prevalence, Structure and Correlates of Anxiety-Depression in boys with an Autism Spectrum Disorder. Res. Dev. Disabil..

[4] White S.W., Oswald D., Ollendick T., Scahill L. (2009). Anxiety in children and adolescents with autism spectrum disorders. Clin. Psychol. Rev..

[5] DeFilippis M. (2018). Depression in Children and Adolescents with Autism Spectrum Disorder. Children.

[6] Mehtar M., Mukaddes N. (2011). Posttraumatic Stress Disorder in individuals with diagnosis of Autistic Spectrum Disorders. Res. Autism Spectr. Disord..

[7] Kenny L., Hattersley C., Molins B., Buckley C., Povey C., Pellicano E. (2016). Which terms should be used to describe autism? Perspectives from the UK autism community. Autism.

[8] American Psychiatric Association (2013). Diagnostic and Statistical Manual of Mental Disorders.

[9] Tierney S., Burns J., Kilbey E. (2016). Looking behind the mask: Social coping strategies of girls on the autistic spectrum. Res. Autism Spectr. Disord..

[10] Winston F., Kassam-Adams N., Garcia-Espana F., Ittenbach R., Cnaan A. (2003). Screening for risk of persistent posttraumatic stress in injured children and their parents. Jama.

[11] Chou W.-J., Wang P.-W., Hsiao R., Hu H.-F., Yen C.-F. (2020). Role of School Bullying Involvement in Depression, Anxiety, Suicidality, and Low Self-Esteem Among Adolescents with High-Functioning Autism Spectrum Disorder. Front. Psychiatry.

[12] Bitsika V., Heyne D., Sharpley C. (2021). Is Bullying Associated with Emerging School Refusal in Autistic Boys?. J. Autism Dev. Disord..

[13] Maïano C., Normand C., Salvas M.-C., Moullec G., Aimé A. (2016). Prevalence of School Bullying Among Youth with Autism Spectrum Disorders: A Systematic Review and Meta-Analysis. Autism Res..

[14] Humphrey N., Hebron J. (2015). Bullying of children and adolescents with autism spectrum conditions: A ‘state of the field’ review. Int. J. Incl. Educ..

[15] Haruvi-Lamdan N., Horesh D., Golan O. (2018). PTSD and autism spectrum disorder: Co-morbidity, gaps in research, and potential shared mechanisms. Psychol. Trauma.

[16] Hull L., Petrides K., Allison C., Smith P., Baron-Cohen S., Lai M., Mandy W. (2017). “Putting on my best normal”: Social camouflaging in adults with autism spectrum conditions. J. Autism Dev. Disord..

[17] Gadow K., Sprafkin J. (2010). Child and Adolescent Symptom Inventory 4R: Screening and Norms Manual.

[18] Gadow K., Devincent C., Pomeroy J., Azizian A. (2005). Comparison of DSM-IV symptoms in elementary school-age children with PDD versus clinic and community samples. Autism.

[19] Weisbrot D., Gadow K., DeVincent C., Pomeroy J. (2005). The presentation of anxiety in children with Pervasive Developmental Disorders. J. Child. Adolesc. Psychopharmacol..

[20] Gadow K., Sprafkin J. (2015). The Symptom Inventories: An Annotated Bibliography.

[21] Sainato D., Goldstein H., Strain P. (1992). Effects of self-evaluation on preschool children’s use of social interaction strategies with their classmates with autism. J. Appl. Behav. Anal..

[22] Capps L., Sigman M., Yirmiya N. (1995). Self-competence and emotional understanding in high-functioning children with autism. Dev. Psychopathol..

[23] Bauminger N., Kasari C. (2000). Loneliness and friendship in high-functioning children with autism. Child. Dev..

[24] Kuusikko S., Pollock-Wurman R., Jussila K., Carter A., Mattila M.-L., Ebeling H., Pauls D.L., Moilanen I. (2008). Social anxiety in high-functioning children and adolescents with Autism and Asperger Syndrome. J. Autism Dev. Disord..

[25] Vickerstaff S., Heriot S., Wong M.L., Lopes A., Dossetor D. (2007). Intellectual ability, self-perceived social competence, and depressive symptomatology in children with high-functioning autistic spectrum disorders. J. Autism Dev. Disord..

[26] Lecavalier L., Gadow K., DeVincent C., Edwards M. (2009). Validation of DSM-IV Model of psychiatric syndromes in children with Autism Spectrum Disorders. J. Autism Dev. Disord..

[27] Bitsika V., Sharpley C., Sweeney J., McFarlane J. (2014). HPA and SAM axis responses as correlates of self- vs parental ratings of anxiety in boys with an Autistic Disorder. Physiol. Behav..

[28] Cohen J. (2004). Statistical Power for the Behavioural Sciences.

[29] Rumball F. (2019). A Systematic Review of the Assessment and Treatment of Posttraumatic Stress Disorder in Individuals with Autism Spectrum Disorders. Rev. J. Autism. Dev. Disord..

[30] Bejerot S., Mortberg E. (2009). Do autistic traits play a role in the bullying of Obsessive-Compulsive Disorder and Social Phobia sufferers?. Psychopathology.

[31] Kerns C., Berkowitz S., Moskowitz L., Drahota A., Lerner M., Newschaffer C. (2020). Screening and treatment of trauma-related symptoms in youth with autism spectrum disorder among community providers in the United States. Autism.

